# Influenza A Virus Coding Regions Exhibit Host-Specific Global Ordered RNA Structure

**DOI:** 10.1371/journal.pone.0035989

**Published:** 2012-04-25

**Authors:** Salvatore F. Priore, Walter N. Moss, Douglas H. Turner

**Affiliations:** Department of Chemistry and Center for RNA Biology, University of Rochester, Rochester, New York, United States of America; CSIR-Institute of Microbial Technology, India

## Abstract

Influenza A is a significant public health threat, partially because of its capacity to readily exchange gene segments between different host species to form novel pandemic strains. An understanding of the fundamental factors providing species barriers between different influenza hosts would facilitate identification of strains capable of leading to pandemic outbreaks and could also inform vaccine development. Here, we describe the difference in predicted RNA secondary structure stability that exists between avian, swine and human coding regions. The results predict that global ordered RNA structure exists in influenza A segments 1, 5, 7 and 8, and that ranges of free energies for secondary structure formation differ between host strains. The predicted free energy distributions for strains from avian, swine, and human species suggest criteria for segment reassortment and strains that might be ideal candidates for viral attenuation and vaccine development.

## Introduction

Influenza A is a (−)sense RNA virus of significant public health concern. Seasonal epidemics result in over 41,400 deaths and 200,000 serious illnesses each year in the United States [1]. More concerning is the ability of Influenza A to cause pandemics. Pandemics are believed to arise because the genome consists of eight single-stranded RNA segments that can reassort from multiple host species to form novel strains to which humans have little or no prior immunity [2]. The 2009 “Swine-flu" pandemic is thought to involve reassortment of genes from avian, swine, and human strains [3]. Because swine cells have surface glycoproteins that are recognized by both avian and human HA protein, they have been proposed as a “mixing vessel" for reassortment [4], [5]. Thus, it is believed that there is a natural species barrier largely preventing direct transmission between avian and human strains [6]. Several studies have identified different aspects of influenza A that restrict host replication range [7], [8], [9], [10], but fundamental factors that differentiate host influenza strains are not well defined.

One potential fundamental species barrier that has not been considered is RNA secondary structure. Many RNA viruses contain functionally important structures that are crucial for efficient replication. Stabilities of such structures will be dependent on temperature, which varies with host species [11], [12]. Bioinformatics approaches have identified several areas of the influenza genome that likely contain conserved RNA secondary structure, especially in the (+)RNA [13], [14], [15], [16], [17]. While most known functional RNA structures are local in nature, extensive base pairing exists throughout the genomes of many (+)RNA viruses [18]. This phenomenon is called Genome-scale Ordered RNA Structure (GORS) and is speculated to be involved in viral persistence and immune system avoidance. Whatever its purpose, it is clear that GORS plays an important role in the function of many viruses because evolutionary constraints on coding regions are apparently increased to maintain extensive base-pairing [18]. A previous study has demonstrated the potential of Influenza A NS1 mRNA to form extensive base pairing and has hypothesized about the role of RNA secondary structure in influenza evolution and pathogenesis [19]. If there were no functional implications, it would be expected that a virus would evolve to have more plasticity in its coding region to adapt to changing immune responses and to acquire drug resistance. The influenza (−)RNA is transcribed to (+)RNA in the host cell without the need for a DNA intermediate. Therefore, it is possible that influenza virus possesses GORS-like features in its coding regions, and that this may be one factor that separates influenza viruses that replicate in different host species. In this paper the GORS acronym is redefined to mean Global Ordered RNA Structure, because both strand orientations are considered. A combination of minimum free energy predictions of native and matched randomized controls is used in this study. Z-scores are calculated from these data to give a measure of how much “excess" stability a sequence possesses than would be predicted for random sequences [20]. A more negative z-score indicates a more stably folded native sequence. Comparisons between predicted thermodynamic stabilities at 37°C of RNA secondary structure of all eight segments in avian, swine and human (+)RNA coding regions and z-scores reveal a pattern in which segments 1, 5, 7 and 8 exhibit host species-specific GORS and free energy distributions. These results contrast with the uniform lack of GORS in (−)RNA.

## Results

### Segments 1(PB2), 2(PB1/PB1-F2), and 3(PA)

**Figure 1:**
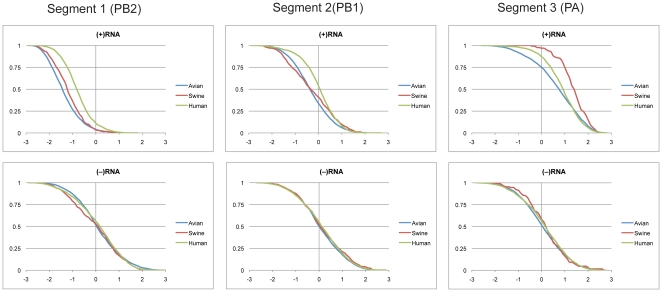
Cumulative distribution plots of z-scores at 37°C for segments 1, 2 and 3. (+)RNA and (−)RNA are shown in the top and bottom panels, respectively. Z-scores are on the x-axis with z-scores predicting more stable RNA secondary structure toward the left of the plot. Fractions of sequences with z-scores greater than or equal to the abscissa are reported on the y-axis. Avian, swine and human sequences are represented by blue, red and green coloring, respectively.

Segments 1, 2, and 3 code for the influenza A polymerase proteins. In addition, segment 2 has additional internal open reading frames that code for a pro-apoptotic peptide, PB1-F2 and a longer N40 protein of unknown function [21], [22]. The cumulative distribution of z-scores (see Materials and Methods) for all three segments is shown in Figure 1. The average predicted z-scores and free energies are presented in Tables 1 and 2, respectively. In the (+)RNA, segment 1 had the most negative average z-score of these segments with avian having the lowest (−1.46), followed by swine (−1.31) and then human (−0.89). Only the distribution of z-scores for segment 1 was significantly shifted into the negative region, while the segment 2 distribution centered around zero and the z-scores for segment 3 were mainly positive. All three segments of (−)RNA had z-scores of approximately zero (Figure 1 and Table 1).

**Table 1:** Average z-scores for (+) and (−)RNA*.

	Avian	Swine	Human
Seg1	−1.46 (0.00)	−1.31 (−0.13)	−0.89 (−0.03)
Seg2	−0.43 (−0.05)	−0.41 (0.03)	−0.09 (0.00)
Seg3	0.54 (−0.04)	1.24 (0.07)	0.76 (0.01)
Seg4	−0.57 (−0.01)	−0.31 (0.00)	−0.13 (0.01)
Seg5	−1.63 (−0.03)	−1.47 (0.00)	−1.29 (−0.01)
Seg6	−0.07 (0.02)	−0.26 (−0.03)	−0.31 (0.02)
Seg7(M1)	−1.29 (−0.01)	−1.14 (−0.02)	−1.61 (0.00)
Seg7(M2)	−0.19 (0.00)	−0.65 (−0.07)	−0.39 (0.10)
Seg8(NS1)	−2.28 (−0.07)	−1.97 (0.00)	−1.54 (−0.03)
Seg8(NEP)	−1.85 (−0.03)	−1.57 (−0.05)	−1.35 (−0.06)

* (−)RNA is reported in parenthesis.

**Table 2:** Average predicted free energies (kcal/mol) at 37°C for (+) and (−)RNA*.

	Avian	Swine	Human
Seg1	−692 (−623)	−669 (−601)	−649 (−591)
Seg2	−636 (−631)	−626 (−616)	−619 (−618)
Seg3	−619 (−609)	−612 (−601)	−599 (−577)
Seg4	−479 (−450)	−465 (−450)	−474 (−461)
Seg5	−494 (−436)	−471 (−417)	−470 (−417)
Seg6	−418 (−372)	−407 (−361)	−411 (−371)
Seg7(M1)	−263 (−240)	−249 (−237)	−260 (−233)
Seg7(M2)	−85 (−65)	−87 (−69)	−84 (−67)
Seg8(NS1)	−233 (−203)	−227 (−198)	−216 (−198)
Seg8(NEP)	−104 (−83)	−99 (−77)	−95 (−76)

* (−)RNA is reported in parenthesis.

**Figure 2:**
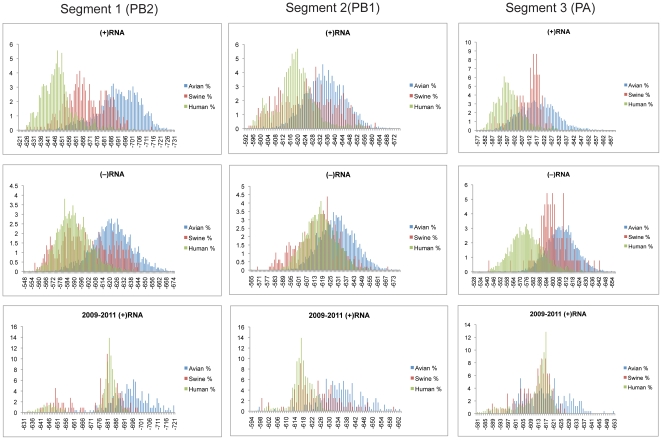
Predicted free energy distributions at 37°C for segments 1, 2 and 3. (+)RNA, (−)RNA, and 2009-present (+)RNA sequences are shown in the top, middle and bottom panels, respectively. Free energy bins are in 1 kcal/mol increments on the x-axis. Percentages of sequences in each bin are reported on the y-axis. Avian, swine and human sequences are represented by blue, red and green coloring, respectively.

Predicted free energy distributions for segments 1–3 are shown in Figure 2. There was a marked distinction between the three host species populations in segment 1. On average, human strains were predicted to be the least stable, with swine occupying an intermediate position between human and avian strains. While segments 1 and 2 were essentially the same length, the predicted average free energies for segment 1 were significantly more stable than for segment 2 for all three species. This difference was also apparent in the lower z-scores in segment 1 (Table 1). The (−)RNA of segment 1 generally had less stable predicted free energies compared to the (+)RNA. Segments 2 and 3 showed no significant change between strand orientations. Therefore, based on the distribution of z-scores, it appears that segment 1 has significant species-specific GORS, but segments 2 and 3 do not.

Another interesting feature of the predicted free energy distributions can be seen with the 2009 pandemic strains. The predicted free energies for the human 2009 H1N1 strains coincide with the main region of overlap between swine and avian strains for segments 1 and 3 (Figure 2). This is not surprising as the phylogenetic origin for these segments was avian [23]. However, this suggests that strains in overlap regions of predicted free energy distributions might be more prone to reassortment than strains at the extremes.

### Segments 4(HA) and 6(NA)

Segments 4 and 6 code for the antigenic proteins that allow influenza A virus to enter and exit host cells. These surface glycoproteins are also major contributors to the immune response in humans. While most influenza A segments have consistently sized coding regions, 4 and 6 have numerous subtypes with different lengths. To compensate for this difference, all sequences for segments 4 and 6 were normalized with respect to length.

**Figure 3:**
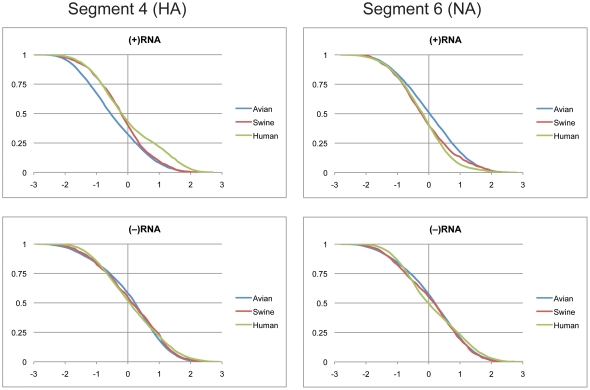
Cumulative distribution plots of z-scores for segments 4 and 6. (+)RNA and (−)RNA are shown in the top and bottom panels, respectively. See Figure 1 for annotations and details.

**Figure 4:**
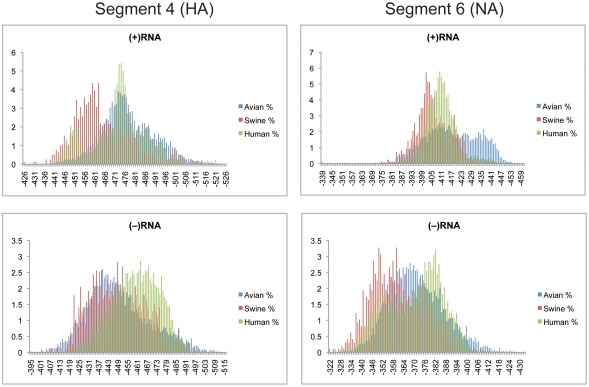
Predicted free energy distributions at 37°C for segments 4 and 6. (+)RNA and (−)RNA are shown in the top and bottom panels, respectively. See Figure 2 for annotations and details.

The cumulative distributions of z-scores for segments 4 and 6 are shown in Figure 3. The average z-scores in the (+)RNA were greater than −0.6 (Table 1) and the distributions did not appreciably fall within the negative range. The distributions of z-scores in the (−)RNA centered around zero (Figure 3). The detailed predicted free energies for segments 4 and 6 are shown in Figure 4. The average free energies were more stable in the (+)RNA than the (−)RNA for all three species (Table 2). The distributions for the (+)RNA were more distinct for each of the species than for the (−)RNA, but both orientations show a high degree of overlap between the three hosts. The slightly negative scores in the (+)RNA could be a sign of local RNA secondary structure present in these segments as previously predicted [17].

### Segment 5 (NP)

Segment 5 codes for the NP protein that binds RNA and serves as a scaffold for ribonucleoprotein production that is critical for viral replication. Segment 5 genes from avian strains are known to cause significant attenuation when crossed with human strains and replicated in mammalian cells both *in vivo* and *in vitro* [8], [9].

**Figure 5:**
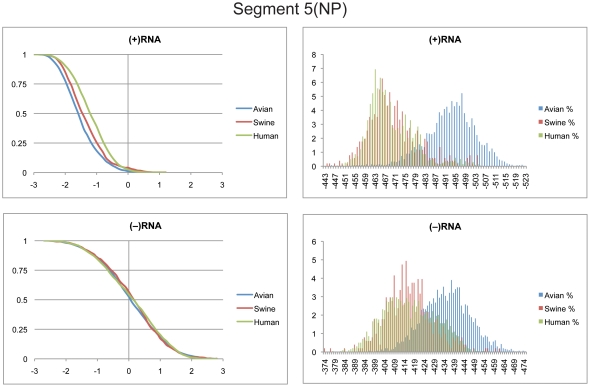
Cumulative and predicted free energy distributions for segment 5. Left: Cumulative distribution plots of z-scores for segment 5 in the (+)RNA (Top) and (−)RNA (Bottom). See Figure 1 for annotations and details. Right: Predicted free energy distributions at 37°C for segment 5 in the (+)RNA (Top) and (−)RNA (Bottom). See Figure 2 for annotations and details.

In light of these findings, it is intriguing that segment 5 had the largest distribution separation of predicted (+)RNA free energies between avian and human strains of any of the influenza segments (Figure 5). The cumulative distribution of z-scores for all three species was significantly shifted to below zero (Figure 5). Thus, segment 5 also appears to possess GORS in the (+)RNA. The distributions of predicted free energies for swine and human strains overlapped almost completely, while avian strains were predicted to be more stable. Sequences more stable than −506 kcal/mol only included avian strains; thus these sequences might be good candidates to test for viral attenuation of human influenza viruses.

The (−)RNA z-scores for segment 5 centered around zero (Figure 5). Predicted free energies in the (−)RNA were significantly less stable with markedly more overlap of the distributions for the different host species relative to the (+)RNA (Figure 5).

### Segments 7 (M1/M2) and 8 (NS1/NEP)

Segments 7 and 8 are both alternatively spliced to code for two proteins each. Segment 7 codes for the M1 and M2 proteins, which are the structural components of the viral membrane and drive new virion assembly [24], [25]. Segment 8 codes for the multi-functional NS1 protein that controls splicing, retention of nuclear RNAs, and other functions [26]. The smaller NEP protein is responsible for the export of viral RNAs out of the nucleus for packaging [27].

**Figure 6:**
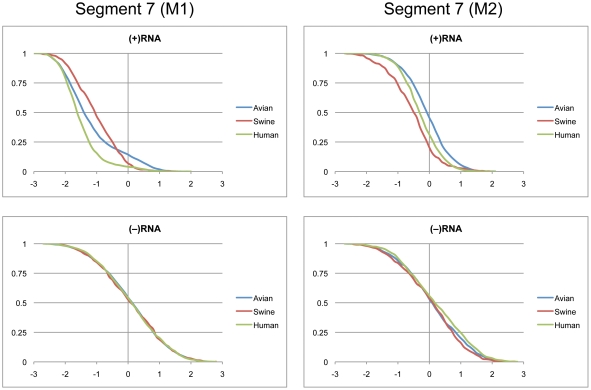
Cumulative distribution plots of z-scores for segment 7 coding regions. M1 and M2 are shown in the left and right panels, respectively. (+)RNA and (−)RNA are shown in the top and bottom panels, respectively. See Figure 1 for annotations and details.

**Figure 7:**
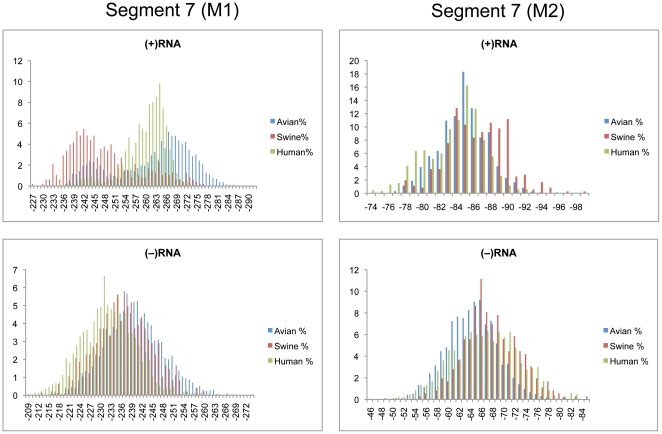
Predicted free energy distributions at 37°C for segment 7 coding regions. M1 and M2 are shown in the left and right panels, respectively. (+)RNA and (−)RNA are shown in the top and bottom panels, respectively. See Figure 2 for annotations and details.

Cumulative distributions for segment 7 z-scores in M1 and M2 coding regions are shown in Figure 6. The distribution for the (+)RNA of M1 were clearly shifted into the negative region, while M2 was only slightly shifted below zero for human and swine sequences. Thus, it appears that M1 possesses GORS and M2 may not. Distributions of z-scores for the (−)RNA of M1 and M2 were centered at zero (Figure 6). The free energy distributions for M1 and M2 are shown in Figure 7. The majority of the swine M1 sequences had the least stable free energies, while human M1 sequences centered near −260 kcal/mol (Table 2). Avian M1 sequences were the most stable on average and displayed a bimodal distribution with peaks at −244 and −268 kcal/mol. As for segment 5 there was an area below −278 where only avian sequences were represented. The predicted free energy distributions for M1 and M2 in the (+) and (−) RNA showed no distinction between the three host species (Figure 7).

**Figure 8:**
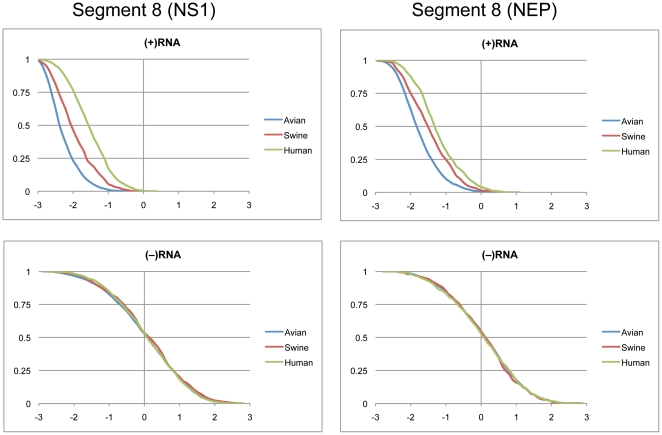
Cumulative distribution plots of z-scores for segment 8 coding regions. NS1 and NEP are shown in the left and right panels, respectively. (+)RNA and (−)RNA are shown in the top and bottom panels, respectively. See Figure 1 for annotations and details.

**Figure 9:**
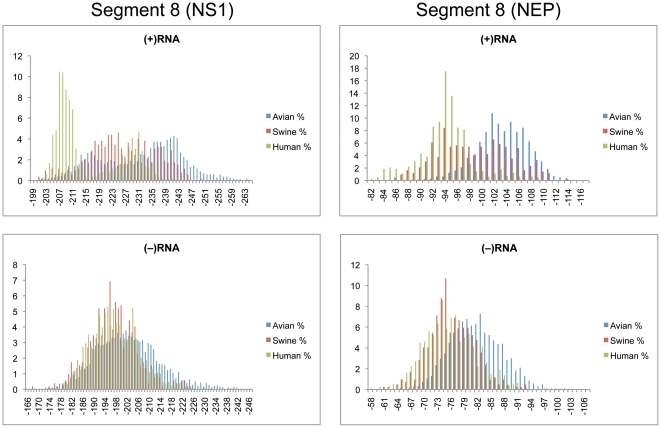
Predicted free energy distributions at 37°C for segment 8 coding regions. NS1 and NEP are shown in the left and right panels, respectively. (+)RNA and (−)RNA are shown in the top and bottom panels, respectively. See Figure 2 for annotations and details.

Cumulative distributions of z-scores for segment 8 NS1 and NEP coding regions are shown in Figure 8. Average z-scores for the (+)RNA were the most negative of all segments for avian and swine strains, and with the exception of segment 7(M1), for human strains as well (Table 1). The z-score distributions for the (+)RNA of both NS1 and NEP were in the negative region, while the (−) RNA was centered around zero. Predicted free energy distributions for human strains of NS1 (+)RNA showed distinct peaks at −207 and −230 kcal/mol (Figure 9). Swine and avian strains were much more widely distributed, but on average swine strains were more stable than human, but less stable than avian strains. There was a region below −251 kcal/mole where only avian strains were represented. The predicted free energy distributions for NEP (+)RNA showed distinct populations for avian and human strains with swine strains widely distributed between the two (Figure 9). Predicted free energy distributions for both NS1 and NEP in the (−)RNA did not show a distinction between host species (Figure 9). In addition, the average free energy in the (−)RNA was significantly less stable when compared to the (+)RNA (Table 2).

## Discussion

This work demonstrates that GORS exists in (+)RNA segments 1, 5, 7, and 8 of Influenza A virus (Figures 1, 5, 6, and 8). For certain segments, this phenomenon is accompanied by distinct distributions of predicted free energies between avian, swine, and human strains. Except for segment 6 and segment 7 (M1 and M2), the most negative average z-scores were for avian strains (Table 1). Avian strains also had the most stable predicted free energy on average, with the exception of segment 7 (M2). It appears that global RNA structure for segments 1, 5, 7(M1), and 8 (NS1 and NEP) may have evolved to have host specificity. Avian, swine and human viruses replicate in distinct environments. Temperatures for the avian gut, and the swine and human respiratory epithelium are 41, 37, and 33°C, respectively [11], [12]. In addition, the pH of the avian gut is presumably much lower than in the human and swine respiratory tract. These changes in ambient temperature and pH are expected to influence the equilibrium of RNA base pairing. For example, segments 7 and 8 are thought to contain regions with temperature dependent equilibria between two folds [17]. It is possible that similar undiscovered equilibria may exist in segments 1 and 5. The original GORS paper postulated the importance of RNA structure to viral persistence and avoidance of host cell immunity [18]. Other host cellular factors such as, protein/mRNA interactions, mRNA decay and translation efficiency could also play a role in directing the host-specific evolution of influenza mRNA stability. Thus, variation of structural stability could be one method of adapting to distinct host environments, but further experiments are needed to clarify the significance of these results. Whatever the reason, the presence of stable structures is likely important to the viral life cycle. While z-score distributions for segments 2, 3, 4, 6, and 7(M2) (+)RNA do not support GORS, there is still the possibility of locally conserved RNA secondary structure as described previously [17].

In contrast, the (−)RNA uniformly lacks stable structure. In every case, the distribution of z-scores in the (−)RNA is centered around zero with no distinction between avian, swine, and human strains. This supports previous results suggesting that conserved secondary structure is heavily favored in the (+)RNA [17]. If the observed bias in stabilities of the (+)RNA were artifacts of sequence nucleotide composition or the free energy prediction model, then similar distributions should be seen in the (−)RNA. This, however, is not the case. The distributions for the (−)RNA are those expected from an ideal negative control for unstructured RNA. As above, the lack of GORS in the (−)RNA does not preclude locally conserved structure, especially in the untranslated regions which were not considered in this study.

The results from this study have several potential applications. Sequence dependent thermodynamics could be a new criterion for gauging the ability of some segments to reassort between host species. This information would be valuable as reassortants can lead to pandemic strains. Segment 1 has very distinct populations between all three host species, while segments 5 and 8(NEP) had relatively large separation between human and avian strains. The areas of overlap on these distributions represent the most likely sequences to reassort, as can be seen with the 2009 H1N1 swine flu (Figure 2). These thermodynamic criteria may also be useful for tracking the evolution of influenza strains.

Segments 5, 7(M1), and 8(NS1) all have areas of predicted free energy distributions where only avian strains are represented. These sequences may be good candidates for attenuation of human strains for vaccine development. While most seasonal influenza vaccines contain no live virus, the development of live attenuated virus vaccines may be desirable because of potential enhanced immunogenicity and commercial production efficiency [28].

It will be important to continue to increase our knowledge of the fundamental species barriers that separate different host strains of influenza virus and the factors that contribute to pandemic reassortment. This and previous studies [13], [14], [15], [16], [17] highlight the need to elucidate the functional implications of global and local RNA structure of influenza A and possibly other viruses that infect multiple hosts.

## Materials and Methods

Coding regions for all unique influenza A mRNAs were downloaded from the NCBI Influenza Virus Resource Page [29]. The data were divided into groups for strains acquired before and after 2009, inclusive. This division was necessary to avoid over representing 2009 H1N1 sequences, which have nearly doubled the size of the NCBI influenza database. Sequences were scanned to remove truncated sequences or those with ambiguous nucleotides. The nearest-neighbor thermodynamic model [30] as implemented by RNAfold [31] was used to predict RNA secondary structure. These calculations were run for both (+)RNA and (−)RNA. All predictions were calculated at 37°C to maximize accuracy and approximate physiological conditions.

Z-scores were calculated for every sequence in both (+)RNA and (−)RNA to test if minimal free energy predictions were more stable than predicted on average for random sequences [20]. Sequences were randomized ten times using the shuffle algorithm in the Simmonics package [32] to maintain dinucleotide frequencies. Free energies for shuffled sequences were calculated as described above.
